# Avian cholera outbreaks threaten seabird species on Amsterdam Island

**DOI:** 10.1371/journal.pone.0197291

**Published:** 2018-05-30

**Authors:** Audrey Jaeger, Camille Lebarbenchon, Vincent Bourret, Matthieu Bastien, Erwan Lagadec, Jean-Baptiste Thiebot, Thierry Boulinier, Karine Delord, Christophe Barbraud, Cédric Marteau, Koussay Dellagi, Pablo Tortosa, Henri Weimerskirch

**Affiliations:** 1 Université de La Réunion, UMR PIMIT (Processus Infectieux en Milieu Insulaire Tropical), CNRS 9192, INSERM 1187, IRD 249, GIP CYROI, Saint Denis, La Réunion, France; 2 Université de la Réunion, UMR ENTROPIE, UR, IRD, CNRS, Saint Denis, La Réunion, France; 3 Centre de Recherche et de Veille sur les maladies émergentes dans l’Océan Indien, GIP CYROI, Sainte Clotilde, La Réunion, France; 4 Centre d’Ecologie Fonctionnelle et Evolutive, CNRS-Université Montpellier UMR 5175, Montpellier, France; 5 Réserve Naturelle Nationale des Terres Australes Françaises, Terres Australes et Antarctiques Françaises, rue Gabriel Dejean, Saint Pierre, La Réunion, France; 6 Centre d’Etudes Biologiques de Chizé, UMR 7372CNRS – Université de La Rochelle, Villiers en Bois, France; Peter Doherty Institute for Infection and Immunity, AUSTRALIA

## Abstract

Infectious diseases may be particularly critical for the conservation of endangered species. A striking example is the recurrent outbreaks that have been occurring in seabirds on Amsterdam Island for the past 30 years, threatening populations of three Endangered seabird species and of the endemic, Critically Endangered Amsterdam albatross *Diomedea amsterdamensis*. The bacteria *Pasteurella multocida* (avian cholera causative agent), and to a lesser extent *Erysipelothrix rhusiopathiae* (erysipelas causative agent), were both suspected to be responsible for these epidemics. Despite this critical situation, demographic trends were not available for these threatened populations, and the occurrence and characterization of potential causative agents of epizootics remain poorly known. The aims of the current study were to (i) provide an update of population trends for four threatened seabird species monitored on Amsterdam Island, (ii) assess the occurrence of *P*. *multocida*, and *E*. *rhusiopathiae* in live birds from five species, (iii) search for other infectious agents in these samples and, (iv) isolate and genotype the causative agent(s) of epizooties from dead birds. Our study shows that the demographic situation has worsened substantially in three seabird species during the past decade, with extremely low reproductive success and declining populations for Indian yellow-nosed albatrosses *Thalassarche carteri*, sooty albatrosses *Phoebetria fusca*, and northern rockhopper penguins *Eudyptes moseleyi*. *Pasteurella multocida* or *E*. *rhusiopathiae* were detected by PCR in live birds of all five investigated species, while results were negative for eight additional infectious agents. A single strain of *P*. *multocida* was repeatedly cultured from dead birds, while no *E*. *rhusiopathiae* could be isolated. These results highlight the significance of *P*. *multocida* in this particular eco-epidemiological system as the main agent responsible for epizootics. The study stresses the urgent need to implement mitigation measures to alter the course of avian cholera outbreaks threatening the persistence of seabird populations on Amsterdam Island.

## Introduction

Infectious diseases are a major concern for wildlife conservation, with strong evidence for their influence in the decline and extinction risk in many populations [1]. Examples include amphibian chytridiomycosis, threatening several species with extinction at local and global scales [2]; canine distemper virus epidemics, causing significant declines in wild canine populations in Serengeti [3], or phocine distemper virus, killing 23,000 North Sea harbour seals *Phoca vitulina* in 1988 and 30,000 in 2002 [4]. Hence, examining the threat posed by such diseases may be particularly critical for the conservation of endangered species [5–8].

Amsterdam Island, a 53 km^2^ volcanic dome located in the southern Indian Ocean (37°5’S, 77°3’E), hosts several emblematic and threatened seabird species. The Critically Endangered Amsterdam albatross *Diomedea amsterdamensis* [9] is endemic to this island, with a unique breeding population comprising less than 200 individuals [10]. Three other seabird species listed as Endangered, also breed on Amsterdam Island: Indian yellow-nosed albatross *Thalassarche carteri* [11], sooty albatross *Phoebetria fusca* [12] and northern rockhopper penguin *Eudyptes moseleyi* [13]. Notably, numbers of Indian yellow-nosed albatross breeding on Amsterdam Is. account for about 70% of the total for this species (Weimerskirch et al. in revision), further supporting the worldwide significance of this island for the conservation of threatened wildlife.

For the past 30 years, the population of yellow-nosed albatrosses has been declining on Amsterdam Island, from 37,000 pairs in the early 1980’s to 27,000 pairs in 2006 [14,15]. This population decrease was first attributed to adult mortality in long-line fisheries, but further investigations suggested that a disease could also cause this population change [15,16]. Long-term monitoring of this population’s demographic parameters showed that lowered adult survival and very low breeding success have both contributed to the population decline until the mid-1990s, but that the subsequent decline was primarily caused by a low fledging success [15]. During the 1996 breeding season, the survey of 100 yellow-nosed albatross nests revealed that 31 chicks died within a few minutes of convulsions, while being apparently healthy and well-fed a few weeks after hatching [16]. Laboratory analyses performed on 21 carcasses showed that 90% of them were infected by the bacteria *Pasteurella multocida* and 10% by the bacteria *Erysipelothrix rhusiopathiae* [16]. *Pasteurella multocida*, and to a lesser extend *E*. *rhusiopathiae*, were thus suspected to induce, every year since the 1980s, extensive chick mortality in yellow-nosed albatrosses on Amsterdam Island, resulting in a low breeding success and decreasing population.

Indeed, *P*. *multocida* is the causative agent of avian cholera, an infectious disease of major economic impact or conservation concern for several animal species worldwide. This bacterial agent is known to infect over 100 species of wild birds and causes recurrent epizootics that can kill tens of thousands of birds in a single event, with little warning [17,18]. Besides, *E*. *rhusiopathiae* causes a variety of diseases worldwide usually named erysipelas, ranging from acute septicemia (with possible death within a few hours) to sub-acute (cutaneous lesions) or chronic diseases (*e*.*g*. polyarthritis, endocarditis) in several animal species, including humans [19,20].

Disease management in wild animal populations threatened with epizootics has recently been attempted and is increasingly being implemented [21]. This framework first requires to delineate the disease impact and extent on host populations, and to characterize the infectious agent(s) responsible for morbidity and mortality [21]. On Amsterdam Island, despite the critical situation of seabirds, no demographic trend has ever been published for the sooty albatross and the northern rockhopper penguin. As for yellow-nosed and Amsterdam albatrosses, the most recent demographic studies were published in 2009 and 2010, respectively [13,15]. Moreover, the potential causative agents of epizootics remain poorly known (occurrence frequency, characteristics) in the yellow-nosed albatross population [16] and have not been examined in others. In this context, this study aims to (i) analyze recent population trends of endangered seabird species on Amsterdam Island, (ii) test for the presence of *P*. *multocida* and/or *E*. *rhusiopathiae* in live birds among these populations, as well as (iii) search for other infectious agents in these samples, and (iv) characterize the causative agent(s) of epizooties from dead birds, through strain isolation and genotyping.

## Materials and methods

### Ethics statement

The animal procedures used in this this study were reviewed and approved by the Comité de l’Environnement Polaire and by the French Ministry of Research (notification number 04939.03).

### Study site and demographic survey

Amsterdam Island is one of the most isolated islands in the world, located 3,400 km from Western Australia, 4,200 km from Eastern Africa and 3,100 km from Antarctica. The island is a volcanic dome rising to 881 m and surrounded by steep cliffs, mostly 20–80 m high but up to >700 m in the south-west (Entrecasteaux cliffs). At its center lies the Plateau des Tourbières, 550–720 m above sea level. The only landing place ashore is in the north, where a permanent scientific station was built in 1949 hosting 20–30 people and catering tourists 2–3 times per year (30–40 people). Five large (over 1,000 g) seabird species breed annually or biennially on Amsterdam Island (see below). Several other seabird species including Antarctic terns *Sterna vittata*, great-winged *Pterodroma macroptera* and grey *Procellaria cinerea* petrels presumably also breed on Amsterdam Island, but in poorly known numbers and locations [22]. After the eradication of feral cattle in 2011, three introduced mammal species remain on the island, namely feral cats *Felis catus*, brown rats *Rattus norvegicus* and house mice *Mus musculus*.

On Amsterdam Island, the main concentration of breeding seabirds is on the Entrecasteaux cliffs where more than 20,000 pairs of Indian yellow-nosed albatross breed with about 400 pairs of sooty albatross and 12,000 pairs of northern rockhopper penguin. However, the very small population (40–50 pairs) of the biennially breeding Amsterdam albatross is found on the Plateau des Tourbières. Standardized counts of breeding pairs and fledged chicks have been conducted on study colonies to track changes in breeding population size and breeding success for these four species. Monitoring of breeding pairs started in 1981 for Amsterdam and yellow-nosed albatrosses (see [13,15] for further details), in 1996 for sooty albatrosses and in 1994 for rockhopper penguins (see [23,24]). Breeding success monitoring also started in 1981 (for Amsterdam and yellow-nosed albatrosses), 1994 (for sooty albatrosses) and 1997 (for rockhopper penguins). In addition, about 60 pairs of brown skuas *Stercorarius antarcticus* currently breed on the Plateau des Tourbières, but so far no long-term monitoring data are available for this species.

In this study, we estimated annual breeding population growth rates *r* using the slope of a log-linear model fitted to the logarithm of the count data for each species. The annual breeding population growth rate was defined as *r* = ln (*λ*), where *λ* = *N*_*t*_/*N*_*t-1*_, and *N*_*t*_ and *N*_*t-1*_ are the abundance of breeding pairs in year *t* and *t-1*, respectively [25]. Trends in time series of breeding success (number of chicks fledged per pair) were tested using linear models fitted to the annual values of breeding success.

### Disease study and sample collection

Fieldwork was carried out on Amsterdam Island from November 2011 to January 2012. Blood samples (1 ml from the tarsus vein), cloacal and oro-pharyngeal swabs were obtained with sterile rayon-tipped applicators (Puritan, Guilford, ME, USA) from apparently healthy birds chosen randomly from the five abovementioned seabird species, except for rockhopper penguins for which only blood and cloacal swabs were collected. Sampled chicks were between one and eight months old and adults were all breeders. Blood collection did not exceed 0.08% of body mass. Swabs were immediately placed in 0.5 ml of RNA NOW^™^ (BIOGENTEX, Seabrook, Texas, USA) and blood samples (drawn using heparinized syringes) were kept in 2 ml tubes for up to four hours until they were centrifuged. Plasma samples were then transferred to cryovials. Red blood cells were stored in 0.5 ml RNA NOW^™^. All samples were stored at -20°C in the field (for four months) and -80°C in the laboratory (for ten months) until analysis.

In addition, five chick carcasses were collected in the field: two sooty (hereafter D1 and D2), two yellow-nosed (D3 and D4) and one Amsterdam (D5) albatrosses. The death of each chick was witnessed except for the Amsterdam albatross, which was found as a desiccated carcass. During the following breeding season (2012–2103), six additional seabird carcasses were collected and included in this study (sooty albatross: one chick, D6; yellow-nosed albatross: one adult, D11, and three chicks, D7, D8 and D12; skua: one chick, D10). The carcasses were immediately frozen and stored at -20°C until analysis (for up to one year).

### Molecular detection of infectious agents in live bird samples

Total nucleic acids extraction was performed following RNA NOW^™^ (BIOGENTEX, Seabrook, Texas, USA) isolation and purification protocol. Total nucleic acids were eluted in a final volume of 60 μl. cDNA were obtained following a previously published protocol [26,27]. Briefly, reverse-transcription was performed on 20 μl of total nucleic acids, using 0.1 μl of random hexamers (Promega, Madison, Wisconsin, USA) and the GoScript^™^ Reverse Transcriptase (Promega, Madison, Wisconsin, USA), under the following thermal conditions: 80°C for 5 min, 25°C for 5 min, 42°C for 60 min and 70°C for 5 min. The obtained cDNA (40 μl) were diluted 1:2 and stored at -20°C until tested.

Polymerase chain reactions (PCR) were performed following published protocols for (1) the detection of *P*. *multocida* [28] and *E*. *rhusiopathiae* [29] on red blood cell and swab samples, (2) the detection of Coronavirus [30], Paramyxovirus [31] and Influenza A virus [32] on cloacal and oro-pharyngeal swabs, and (3) the detection of *Rickettsia* [33], Flavivirus [34] and Haemosporidia (*Haemoproteus*, *Plasmodium*, *Leucocytozoon*; [35] on red blood cell samples. These infectious agents were tested because they have been described in seabirds, notably in the Indian Ocean [27,36–39] and the Southern Ocean [40,41].

The ABsolute Blue qPCR Low ROX Mix (Thermo Fisher Scientific, Surrey, UK) was used for Real-Time PCR detection (Coronavirus, Paramyxovirus and Influenza A virus), the ABsolute Blue qPCR SYBR Low ROX Mix (Thermo Scientific, Surrey, UK) for Real-Time SYBR PCR detection (Flavivirus), and GoTaq Hot Start Green Master Mix 2X (Promega, Madison, Wisconsin, USA) for end-point (*P*. *multocida*, *E*. *rhusiopathiae*, *Rickettsia*) or semi-nested quantitative PCR (Haemosporidia) in final volumes of 25 μl containing 5 μl of cDNA. PCRs were carried out in a BIO-RAD CFX96 Touch^™^ thermocycler (BIO-RAD, Hercules, California, USA). Amplification products were analysed by electrophoresis on agarose gels stained with 2% GelRed^™^ (Biotium, Hayward, California, USA).

### Bacterial isolation from seabird carcass samples

Samples of lung, liver, heart and bone marrow from all carcasses were collected (except for D5, for which only the bone marrow could be tested due to its decomposition state) and crushed in 1 ml of Lysogeny broth. For each bird, 100 μl of each organ homogenate was streaked onto a blood agar plate (blood agar base supplemented with 5% defibrinated sheep blood; BIO-RAD, Hercules, California, USA) and incubated at 37°C for 24 hr. On each plate, all bacterial morphospecies were visually differentiated and subsequently tested by PCR for *P*. *multocida* [28] and *E*. *rhusiopathiae* [29]. In addition, 16S locus was amplified for each morphospecies using bacterial generic primers (primers 27F and 1492R). Amplicons were subsequently submitted to direct Sanger sequencing (Genoscreen, Lille, France) allowing the identification of bacteria at the genus/species level http://blast.ncbi.nlm.nih.gov/Blast.cgi using GenBank BLASTN 2.2.29+ [42]. Lastly, for each morphospecies identified as *P*. *multocida* in 2011–2012, 18 randomly selected bacterial colonies were further tested for *P*. *multocida* by PCR [28] to validate our visual identification.

### *Pasteurella* genotyping

One isolate of *P*. *multocida* was randomly selected from each culture-positive carcass. Six isolates (D2, D3, D4, D8, D11, D12) were thus genotyped using a set of seven housekeeping genes (*adk*, *est*, *gdh*, *mdh*, *pgi*, *pmi*, and *zwf*), following the multi-locus sequence typing (MLST) scheme developed by [43]. Amplicons were submitted to direct Sanger sequencing (Genoscreen, Lille, France) and nucleotide sequences were edited using CLC Sequence Viewer version 7.7.1 (CLC Bio, Aarhus, Denmark). Allele numbers and sequence types (ST) were identified by comparison with reference strains deposited in the *P*. *multocida* MLST database (http://pubmlst.org/pmultocida/) sited at the University of Oxford [44]. A minimum-spanning tree was built using goeBURST Full MST algorithm (PHYLOViZ 1.1, 2014), by concatenating all seven MLST genes found in various hosts worldwide. *Pasteurella multocida* nucleotide sequences obtained in this study are available from GenBank under the accession numbers MF040795 to MF040801.

## Results

### Demographic study

**Fig 1:**
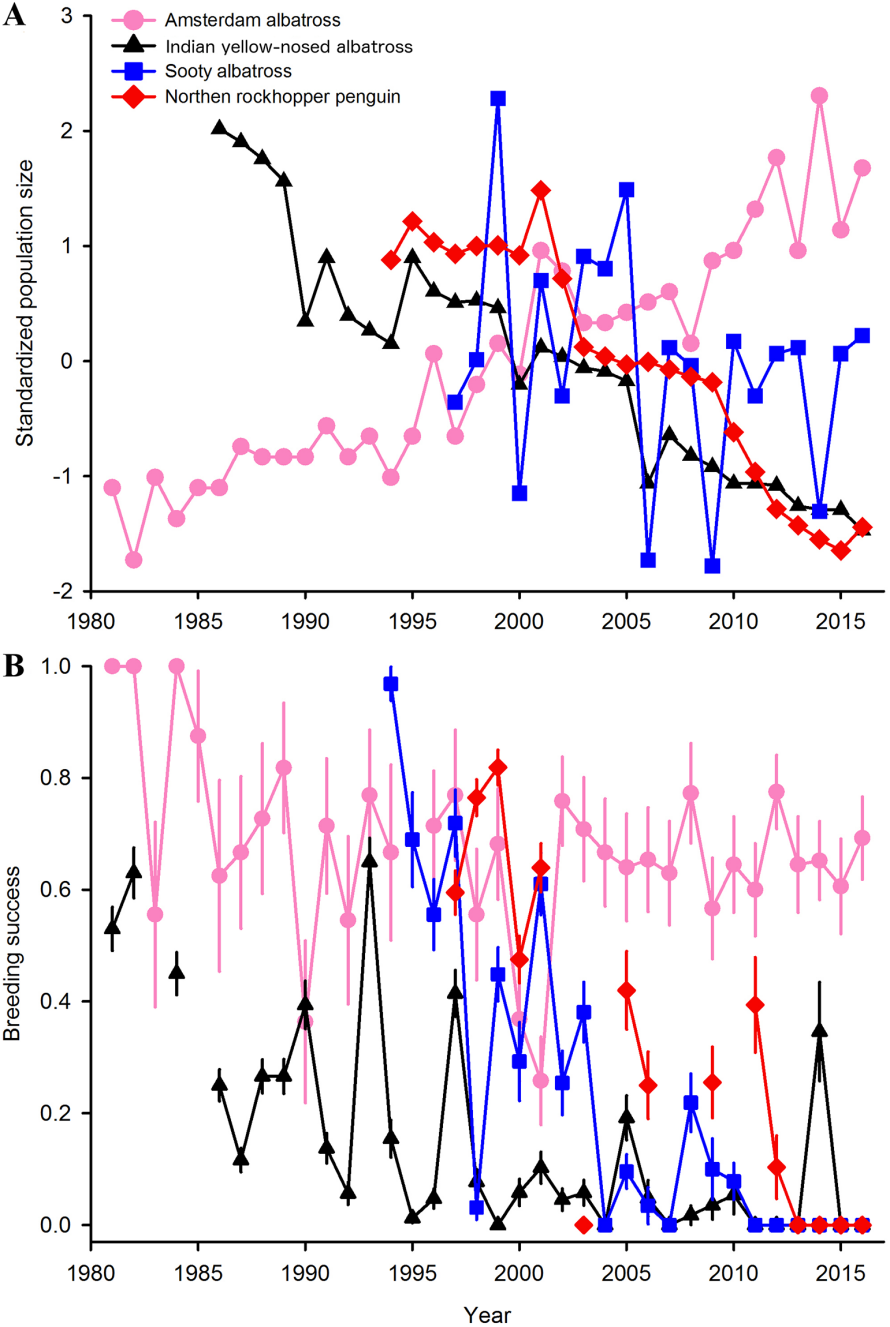
Breeding population trends of four seabird species at Amsterdam Island since 1981. Changes in (a) standardized population sizes and (b) breeding success.

Changes in breeding population sizes differed among seabird species (Fig 1a), with a marked overall decrease for three of the four monitored species. While the small breeding population of Amsterdam albatross has increased since the early 1980’s at an annual rate of 0.0605, resulting in a 760% increase over 1981–2016, the monitored breeding population of Indian yellow-nosed albatrosses has been dramatically declining at an annual rate of -0.0569, resulting in a 86.6% decrease through 1981–2016. A comparable decrease (annual rate: -0.0777) was recorded for breeding northern rockhopper penguins, resulting in a 81.9% decrease during 1994–2016, while the small numbers of breeding sooty albatrosses have been variable, although slightly declining at an annual rate of -0.0094 resulting in a 17.4% decrease through 1996–2016.

The annual breeding success also differed among species (Fig 1b). Breeding success was high on average for the Amsterdam albatross (mean ± sd: 0.677 ± 0.160), except for a few years when high chick mortalities were recorded (1990, 2000 and 2001), with a slight declining trend (slope = -0.005 ± 0.002, P = 0.056). By contrast, the breeding success of Indian yellow-nosed albatrosses was low (0.159 ± 0.193), declining during the 1980’s (slope = -0.012 ± 0.003, P < 0.001), and eventually remaining very low, with the exception of a few years (1990, 1993, 1997 and 2014) with higher success. Breeding success for the sooty albatross was 0.238 ± 0.292 and also declining (slope = -0.035 ± 0.006, P < 0.001), with recurrent very low values including total breeding failures during the very last years (2011–2016). The breeding success of the northern rockhopper penguin was 0.314 ± 0.297 and also declined throughout the period (slope = -0.037 ± 0.007, P < 0.001), with complete breeding failure within recent years (2013–2016).

### Molecular detection of infectious agents in live birds

**Fig 2:**
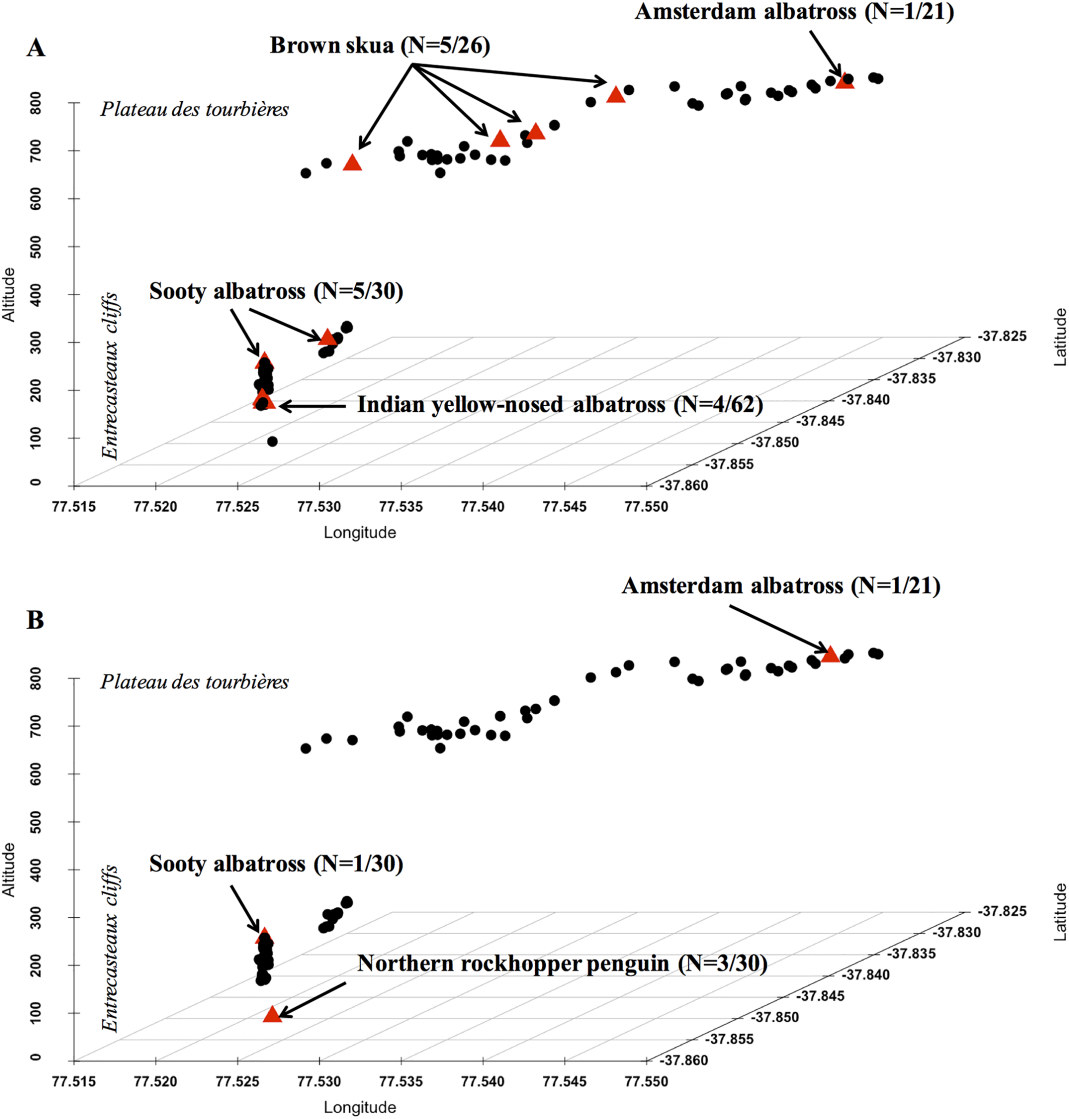
Spatial locations of the individual seabirds which tested positive (red triangles) or negative (black dots) for (a) *Pasteurella multocida* and (b) *Erysipelothrix rhusiopathiae* on Amsterdam Island. Horizontal axes represent longitude (x-axis) and latitude (z-axis); the vertical axis (y-axis) is the elevation of the nests above sea level. The Amsterdam albatross and the brown skua breed on the Plateau des Tourbières, while the Indian yellow-nosed and sooty albatrosses and the northern rockhopper penguin breed in the Entrecasteaux cliffs.

Coronavirus, Paramyxovirus and Influenza A virus were not detected in cloacal and oro-pharyngeal swabs, nor were Flavivirus, *Rickettsia*, and Haemosporidia (*Haemoproteus*, *Plasmodium*, *Leucocytozoon*) in red blood cell samples. All samples (i.e. blood and swabs) were also screened for *P*. *multocida* and *E*. *rhusiopathiae* DNA (Table 1). Fifteen samples tested positive for *P*. *multocida* and five for *E*. *rhusiopathiae*, all corresponding to cloacal or oro-pharyngeal swabs. None of the samples tested positive for both *P*. *multocida* and *E*. *rhusiopathiae*, nor was any individual bird positive for both cloacal and oro-pharyngeal swabs. The spatial distribution of positive samples on the island was very broad (Fig 2). *Pasteurella multocida* was detected in swabs collected from one Amsterdam albatross chick, four Indian yellow-nosed albatross chicks, five adult sooty albatrosses, and five adult brown skuas (Table 1). *E*. *rhusiopathiae* DNA was found in swabs obtained from one Amsterdam albatross chick, one adult sooty albatross, and from three adult northern rockhopper penguins.

**Table 1:** Results of PCR detection of *Pasteurella multocida* and *Erysipelothrix rhusiopathiae* in biological samples collected between November 2011 and January 2012, from five seabird species breeding on Amsterdam Island. CS: cloacal swabs, OS: oro-pharyngeal swabs, and RBC: red blood cell samples. Values are percentages of positive samples (95% confidence intervals).

Species	Age	Number of samples collected			*Pasteurella multocida*			*Erysipelothrix rhusiopathiae*		
		CS	OS	RBC	CS	OS	RBC	CS	OS	RBC
Amsterdam albatross	Chicks	21	21	21	0	4.8 (0–13.9)	0	0	4.8 (0–13.9)	0
Indian yellow-nosed albatross	Chicks	12	12	12	33.3 (6.7–60.0)	0	0	0	0	0
	Adults	50	50	50	0	0	0	0	0	0
Sooty albatross	Adults	30	30	30	16.7 (3.3–30.0)	0	0	0	3.3 (0–9.8)	0
Brown skua	Chicks	10	10	10	0	0	0	0	0	0
	Adults	16	16	16	6.3 (0–18.1)	25.0 (3.8–46.2)	0	0	0	0
Northern rockhopper penguin	Adults	30	0	31	0	-	0	10 (0–20.7)	-	0
Total		169	139	170	5.9 (2.4–9.5)	3.6 (0.5–6.7)	0	1.8 (0–3.8)	1.4 (0–3.4)	0

### Bacterial isolation using tissues from bird carcasses

For samples collected in 2011–2012, tissues from three chick carcasses (one sooty albatross and two Indian yellow-nosed albatrosses) yielded positive *P*. *multocida* cultures, while tissues from the other sooty albatross chick and the Amsterdam albatross chick were both negative through culture (Table 2). A single morphospecies was recorded on Petri dishes plated with heart (26, 924 and 474 colonies from D2, D3 and D4 carcasses), lung (872, 1,744 colonies from D3 and D4 carcasses), bone marrow (eight colonies from D4 carcass) and liver samples (1,188 colonies from D4 carcass). This morphospecies was identified as *P*. *multocida* (18 of 18 randomly selected colonies on each plate and all eight colonies for D4 carcass bone marrow). Cultures attempted from the lung of D2 carcass and from bone marrow of the D3 carcass led to a bacterial lawn composed, based on 16S sequencing, of four bacterial species, namely *P*. *multocida* and three common Enterobacteria species: *Enterococcus faecalis*, *Enterobacter ludwigii* and *Escherichia coli*.

**Table 2:** *Pasteurella multocida* positive cultures obtained from heart, lung, bone marrow and liver from 11 seabird carcasses. All culture plates harbored a single, identical morphospecies, except for those two indicated by an asterisk.

Carcass number	Species	Status	Breeding season	*P*. *multocida* positive tissues
D1	Sooty albatross	Chick	2011–2012	-
D2	Sooty albatross	Chick	2011–2012	Heart, lung*
D3	Indian yellow nosed albatross	Chick	2011–2012	Heart, lung, bone marrow*
D4	Indian yellow nosed albatross	Chick	2011–2012	Heart, lung, bone marrow, liver
D5	Amsterdam albatross	Chick	2011–2012	-
D6	Sooty albatross	Chick	2012–2013	-
D7	Indian yellow nosed albatross	Chick	2012–2013	-
D8	Indian yellow nosed albatross	Chick	2012–2013	Heart, lung, liver
D10	Brown skua	Chick	2012–2013	-
D11	Indian yellow nosed albatross	Adult	2012–2013	Heart, lung, bone marrow, liver
D12	Indian yellow nosed albatross	Chick	2012–2013	Heart, lung, bone marrow, liver

* indicates that other bacterial species were identified in culture plates: *Enterococcus faecalis*, *Enterobacter ludwigii* or *Escherichia coli*.

Similarly, in 2012–2013, *P*. *multocida* was isolated from tissues of three of the six carcasses (two chicks and one adult Indian yellow-nosed albatross), while one brown skua, one sooty albatross, and one Indian yellow-nosed albatross were negative (Table 2). A single morphospecies (identical to that identified as *P*. *multocida* in the 2011–2012 samples) was found on Petri dishes with high bacterial load (1:100 dilution was necessary to obtain isolated colonies) that was further confirmed as *P*. *multocida* by PCR.

### *Pasteurella multocida* genetic characterization

**Fig 3:**
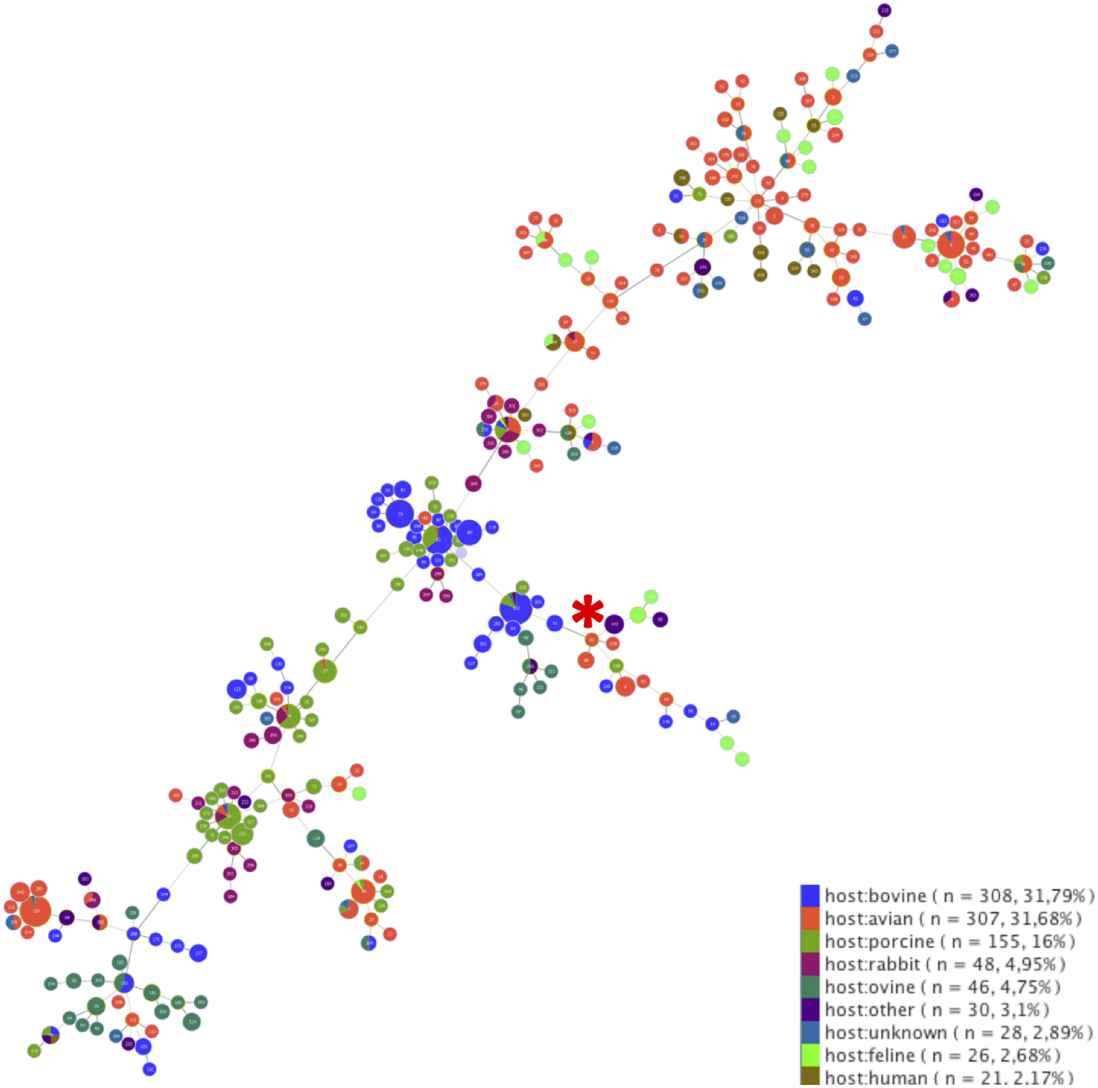
Minimum-spanning tree of *Pasteurella multocida* sequence types (STs) based on the MLST Scheme (http://www.pubmlst.org/pmultocida/). Each circle represents a ST (reference numbers are inside circles). ST61 is specified by a vertical black arrow. The color code indicates the animal origin of sequenced isolates available in the database, n represents the number of these isolates for each animal group.

All six *P*. *multocida* isolates (one isolate randomly selected for each positive carcass: D2, D3, D4, D8, D11, D12) were identical based on the seven sequenced housekeeping genes (100% nucleotide identity). Comparison with reference sequences available in the MLST database revealed that a single *P*. *multocida* allelic profile was found on Amsterdam Island corresponding to sequence type (ST) 61. This particular ST has been previously identified in a duck, in Denmark, in 1985 (isolate number 29135–1). It belongs to *P*. *multocida* group A (*P*. *multocida multocida*). The genetic relationships of this ST with other lineages are shown on a minimum-spanning tree built using data from 969 isolates available on *Pasteurella multocida* MLST database. ST61 can be considered as a founder group since this lineage has evolved towards several other STs found in multiple hosts (Fig 3).

## Discussion

### Population trends and breeding success

Amsterdam Island is a critical breeding ground for several threatened seabird species. Our study shows that the demographic situation has worsened substantially for three species since previous reports published 13 years ago [16]. In Indian yellow-nosed albatrosses, the overall breeding success has remained below 15% for the past 15 years, and the occasional higher success years observed during the 1980’s and 1990’s have become scarcer. Sooty albatrosses have suffered an abrupt decline in reproductive success between 1995 and 2005 with total breeding failure since 2011. The situation has also worsened for the northern rockhopper penguin, with zero fledging since 2013 and a nearly 82% population decrease over the past 22 years. In contrast, the Amsterdam albatross breeding population has increased and breeding success has been high on average, apart from three years (1990, 2000 and 2001) of high chick mortality. As *P*. *multocida* was detected in this species and their nests are situated only 2–3 km away from the cliffs where Indian yellow-nosed albatrosses, sooty albatrosses and northern rockhopper penguin breed, we would expect a higher impact of outbreaks on the Amsterdam albatross population. In this species, nests are widely interspersed, with very low density over the plateau des Tourbières (~0.2 nest/ha) contrary to high nest densities in colonies of northern rockhopper penguin (~3,500 nests/ha) or Indian yellow-nosed albatrosses (~3,100 nests/ha). It is possible that this low nest density limits the spreading of infectious diseases and hence its impact on the population [18]. However, a comprehensive exploration of the epizootiology of outbreaks occurring on Amsterdam Island is required to estimate the intensity of threat for this Critically Endangered species.

### Occurrence of *P*. *multocida*, *E*. *rhusiopathiae* and other infectious agents in apparently healthy birds

None of the eight targeted infectious agents beside *P*. *multocida* and *E*. *rhusiopathiae* (Coronavirus, Paramyxovirus, Influenza A, Flavivirus, *Rickettsia*, *Leucocytozoon*, *Pasmodium*, or *Haemoproteus*) was detected on the 170 tested birds. However, strong temporal dynamics has been reported for these infectious agents in wild birds [45], suggesting that the detection failure may reflect an absence of targeted agents at time of bird sampling. Hence, although the circulation of these agents cannot be definitively ruled out on the basis of this study, there is no evidence suggesting that they may be involved in massive mortalities of Indian yellow-nosed and sooty albatross chicks. In contrast, PCR screening confirmed the circulation of *P*. *multocida* and *E*. *rhusiopathiae*, both previously reported in 1995–1996 [16]. At least one individual of each study species carried one of these two bacteria, but no coinfection was detected. In Amsterdam albatrosses, both bacteria were detected although with low prevalence (9.5%, 95% confidence interval: 0–22.1). One live chick tested positive for *P*. *multocida* and one other for *E*. *rhusiopathiae*. Surprisingly, no adult Indian yellow-nosed albatross (out of 50 tested) tested positive for any of these bacteria at the time of sampling. In contrast, a high prevalence of shedding adult brown skuas was found for *P*. *multocida*, although this may be affected by our limited sample size (16 tested birds). Skuas are predator and scavenger birds that frequently visit the Indian yellow-nosed albatross, sooty albatross and northern rockhopper penguin colonies in search of unattended eggs and chicks. Moreover, skuas and Amsterdam albatrosses breed in the same area (Plateau des Tourbières, Fig 2). Predation or scavenging of infected animals can play a role in the epizootiology of avian cholera [17,18]. Several scavenger species have been reported to become chronically infected with *P*. *multocida*, which could greatly facilitate their ability to spread the bacteria to new sites [17,46,47]. It is thus conceivable that skuas are involved in *P*. *multocida* inter-species transmission on Amsterdam Island and represent a risk for the Critically Endangered Amsterdam albatross, although such hypothesis requires thorough investigation.

### Identification and characterization of disease agents

Almost pure, high density *P*. *multocida* colonies grew in cultures from tissues sampled from six carcasses (out of 11), which is strong evidence for this agents’ implication in the death of these animals. *Pasteurella multocida* can infect a variety of avian species and individual susceptibility to this bacterium is highly variable among avian species [18]. However, acute disease onset is common for avian cholera and has been reported in a wide variety of domestic and wild birds globally [17]. In the wild, the disease primarily affects densely aggregating bird species such as waterfowl and seabirds [46]. Several severe outbreaks of avian cholera in seabirds of the Southern Ocean have been previously reported [47–51]. Our study and the previous report from Weimerskirch [16] suggest that avian cholera could have become endemic within Amsterdam Island seabirds since the 1980’s. These outbreaks may continue to significantly affect population trends and thus threatens the persistence of these seabird populations.

Although tissues from some adult Indian yellow-nosed albatross carcasses were positive for *P*. *multocida* culture, massive deaths recorded in chicks indicate that infection with this bacterium is likely more prevalent in chicks than in adults (this study and [16]). Age-dependent susceptibility to avian cholera varies among bird species. On Amsterdam Island, chicks appear more susceptible than adults, while opposite trends have been reported in several duck species [17]. Factors accounting for this age-and species-dependent susceptibility remain unknown. Little is known about factors influencing immunity and resistance or tolerance of seabird hosts exposed to infectious agents. In the house finch *Haemorhous mexicanus*-*Mycoplasma gallisepticum* system, complex interactions between variability in the virulence of *Mycoplasma* strains and in the immune response of host individuals and populations have been suggested to explain differential epidemiological dynamics [52]. This topic would notably require more attention on Amsterdam Island, especially as strong differential effects appear to exist between albatrosses and skuas, but also between age classes.

We could not isolate *E*. *rhusiopathiae* from any of the dead animals sampled herein. However, this pathogen was isolated in two out of 21 dead chicks collected in 1996, a year with a low recorded chick mortality [16]. The potential involvement of *E*. *rhusiopathiae* in bird morbidity and mortality on Amsterdam Island remains unknown.

### Molecular typing of *P*. *multocida* and potential introduction scenarios

Multi-locus sequence typing (MLST) showed that all Amsterdam Island *P*. *multocida* isolates corresponded to a single ST previously reported from a duck in Denmark in 1985 (sequence type ST61; [53]). Recent studies on the phylogenetic evolution of *P*. *multocida* have shown that this ST belongs to *P*. *multocida* group A (*P*. *multocida multocida*), a lineage with large host diversity in birds and mammalians [54]. *Pasteurella m*. *multocida* appears to be the most common subspecies isolated from wild birds [18]. The absence of genetic variation within the housekeeping genes included in the MLST scheme precludes more detailed evolutionary analysis, but allows introduction scenarios.

Natural introduction through contacts of adult birds with infected species or environments might occur off the coasts of South Africa or Australia, where albatrosses and skuas from Amsterdam can be found during the non-breeding period [55,56]. Scavenger species have been suspected to initiate avian cholera outbreaks by spreading *P*. *multocida* to new places [17,46,47]. However, the absence of genetic diversity in bacterial strains isolated from distinct seabird species over two successive breeding seasons in this study, rather supports a single introduction event followed by the endemisation of this bacterial pathogen on Amsterdam Island.

The introduction of the specific *P*. *multocida* strain that has likely been causing avian cholera outbreaks over the last three decades may be anthropogenic. Firstly, chickens were introduced in the early 1960’s and raised until 2007 in an open-air enclosure on Amsterdam as a food resource for the base. Contacts between poultry and brown skuas have been observed during that period with skuas occasionally landing within the poultry enclosure searching for food, and one can reasonably assume that this may have led to the initial spread of *P*. *multocida* on the island. Secondly, humans may also have contributed to the spread of these infectious agents throughout the island when commuting and/or conducting research activities into seabird colonies. Since 2011, stringent biosecurity measures have been set-up and implemented to limit access to the seabird breeding areas and hence reduce the likelihood of pathogen spread through human activities. Thirdly, mammals are known to host and transmit *P*. *multocida* to poultry [57,58], so one could expect the introduced brown rat to play a role in the local circulation of this pathogen. Brown rats were accidently introduced at least one century ago by boats onto the island, where they now roam, including within seabird colonies. Although we cannot rule out the possibility that they may be involved in the transmission and/or maintenance (see below) of *P*. *multocida*, the absence of bird die-offs in the 1980’s suggests that rats may not be at the origin of the introduction of these infectious agents on the island.

### Disentangling *P*. *multocida* maintenance and transmission on Amsterdam Island

Our results show that the demographic situation of the seabird community on Amsterdam Island has worsened over the past decade, with extremely low reproductive success and declining population trends for Indian yellow-nosed albatrosses, sooty albatrosses and northern rockhopper penguins. Molecular and bacteriological analyses strongly suggest that *P*. *multocida* ST61 is responsible for significant and recurrent outbreaks in Indian yellow-nosed and sooty albatross chicks, but not for the northern rockhopper penguin for which further investigations are required. No dead rockhopper penguin was found in the field during our stay on the island, and *P*. *multocida* was not detected in live penguins while we did detect *E*. *rhusiopathiae* DNA.

The need to control a disease in free-living populations is often justified by conservation concerns. In this study, the identification of *P*. *multocida* as the cause of several epizootics paves the way for further investigations and the subsequent implementation of specific control actions. The mechanisms of bacterial inter-season maintenance and the elucidation of transmission pathways during the breeding seasons must be urgently assessed. The putative role of brown rats as maintenance hosts has been extensively documented in other situations [58–60] and rats could actually be maintaining the bacteria between breeding seasons while seabirds are absent from the island [55,56]. In addition, rats and seabird chicks are both preyed by brown skuas, which may transmit the bacteria from rodents to albatrosses and between albatrosses. Sampling and screening of rats and skuas during and after the breeding season is thus urgently needed to assess their roles in bacterial maintenance and transmission. Lastly, abiotic reservoirs such as wetlands have also been investigated in other *Pasteurella* outbreaks in wild birds [61]. Hence ponds, soil and nests within the colonies could also serve as a source of infection and should be explored.

If brown rats were identified as a major reservoir, then a control of this invasive species would be expected to have a major positive impact on seabird population dynamics [62]. In addition, *P*. *multocida* isolates obtained herein allowed the development of an experimental vaccine, the efficacy of which was evaluated in a controlled study [63]. Any mitigation measure should be eventually included within a proactive strategy aiming at controlling disease outbreaks whose demographic consequences threaten the persistence of seabirds on Amsterdam Island, including flagship species such as albatrosses.
